# Molecular Insights into HR-HPV and HCMV Co-Presence in Cervical Cancer Development

**DOI:** 10.3390/cancers17040582

**Published:** 2025-02-08

**Authors:** Rancés Blanco, Juan P. Muñoz

**Affiliations:** 1 Independent Researcher, Av. Vicuña Mackenna Poniente 6315, La Florida 8240000, Chile; 2 Laboratorio de Bioquímica, Departamento de Química, Facultad de Ciencias, Universidad de Tarapacá, Arica 1000007, Chile

**Keywords:** human papillomavirus, HPV, human cytomegalovirus, HCMV, oncoproteins, cervical cancer

## Abstract

Background: Cervical cancer remains a significant health concern worldwide and the primary cause of cancerous cervical lesions is the infection with high-risk human papillomavirus (HR-HPV). However, emerging evidence suggests that HR-HPV infection alone is insufficient for cancer development, and other co-factors may contribute to cervical carcinogenesis. Human cytomegalovirus (HCMV), a common herpesvirus frequently detected in cervical cancer samples, has demonstrated oncogenic potential. Objectives: This review aims to explore the molecular interactions between HR-HPV and HCMV in promoting cervical cancer progression. Methods. A comprehensive search was conducted in PubMed and Google Scholar, focusing on articles examining the role of HCMV in cervical tissues and/or cells, selected based on relevance and significance. Results: The reviewed literature indicates that HCMV and HR-HPV share several oncogenic mechanisms that could drive cervical cell transformation. Conclusions. Both viruses may synergistically promote cervical epithelial transformation and tumor progression in multiple ways. HR-HPV may facilitate HCMV entry by increasing host cell receptors essential for viral attachment. Additionally, HR-HPV and HCMV may cooperatively disrupt cellular processes, enhancing carcinogenesis. Both viruses may also modulate the local immune environment, enabling immune evasion and lesion persistence. However, further in vitro and in vivo studies are required to validate these hypotheses.

## Simple Summary

High-risk human papillomavirus (HR-HPV) infection is recognized as the primary cause of nearly all cervical cancer cases. However, the evidence suggests that HPV alone may not be sufficient to drive cervical carcinogenesis, pointing to additional co-factors. Notably, recent findings highlight that human cytomegalovirus (HCMV), a herpesvirus commonly detected in cervical lesions, may amplify cancer-related processes. This review examines the current literature on the possible synergistic role of HR-HPV and HCMV in cervical cancer development. The literature reviewed suggests that HCMV could work alongside HR-HPV to disrupt cellular stability, weaken immune defenses, and promote ongoing malignant transformation, potentially accelerating cervical carcinogenesis.

## 1. Introduction

Uterine cervix cancer remains a significant global health concern. In 2022, an estimated 661,021 new cases and approximately 348,189 deaths were attributed to this disease worldwide [1]. Although the overall incidence of cervical cancer has declined or stabilized in many regions due to widespread screening and early intervention, there has been an alarming rise in incidence among younger women. Factors such as early initiation of sexual activity and having multiple sexual partners contribute to the elevated incidence, as they increase the risk of human papillomavirus (HPV) infection [2]. Importantly, HPV vaccination has shown to be highly effective in preventing cervical cancer, with significant reductions in both HPV infections and preinvasive lesions observed, particularly among young women vaccinated before age 13 [3,4].

The primary cause of pre-cancerous and cancerous cervical lesions is the infection with high-risk or oncogenic HPV (HR-HPV) types [5]. Although only 12 genotypes are currently classified as carcinogenic, the majority of cervical cancer cases are attributed to HPV types 16 and 18 [6]. HPV is typically transmitted through sexual contact and leads to the development of squamous intraepithelial lesions (SILs). However, most of these lesions resolve within 6–12 months due to the immune response, and only ~3% progress to cervical precancer/cancer within 7 years [7,8].

Since only a small proportion of SILs persist and progress to cancer, it is suggested that persistent HR-HPV infection is necessary but not sufficient for the development of cervical cancer [9]. Therefore, several authors have suggested that HPV-mediated cervical carcinogenesis seems to need the presence of additional co-factors [10,11]. Some conditions have been associated with an increased risk of cervical cancer, including early sexual activity, multiple sexual partners, high parity, long-term oral contraceptive use, smoking, and co-existing sexually transmitted infections, such as HIV [12,13,14,15]. Notably, studies indicate that co-infection with other viruses—such as Epstein–Barr virus (EBV), Merkel cell polyomavirus (MCPyV), herpes simplex virus type 2 (HSV-2), and particularly human cytomegalovirus (HCMV)—may play a critical role in cervical cancer progression [16,17,18,19,20,21,22]. Specifically, infections with EBV (OR = 4.01, 95% CI: 1.87–8.58), HSV-2 (RR = 2.79, 95% CI: 1.31–5.96), and HCMV (OR = 3.23; 95% CI: 1.12–9.34) have been associated with a significantly increased risk of high-grade squamous intraepithelial lesions (HSIL) or cervical cancer development [22,23,24].

HCMV, also known as human herpesvirus-5 (HHV-5), is a ubiquitous beta-herpesvirus that infects a significant portion of the global population, often establishing lifelong latency with periodic reactivation, particularly in immunocompromised individuals [25]. While typically asymptomatic in healthy individuals, HCMV has been increasingly recognized for its potential role in various cancers, including cervical cancer, due to its ability to modulate host immune responses, promote chronic inflammation, and interfere with cellular processes such as apoptosis and DNA repair [26,27,28,29].

HCMV genomes or gene products have been detected in several cancers, such as malignant glioma, cervical carcinoma, Kaposi’s sarcoma, and breast cancer [30,31,32,33,34]. Emerging evidence suggests that HCMV may contribute to tumorigenesis either directly, through viral gene expression within infected cells, or indirectly, by altering the microenvironment to favor malignant transformation [35,36]. A meta-analysis by Marinho-Dias and Sousa reported an HCMV infection prevalence of 36.5% in HPV-positive cervical samples. Notably, HCMV infection rates increased from normal cervicitis (17.4%) and low-grade squamous intraepithelial lesions (LSIL) (28.0%) to HSIL (39.7%) and in situ/invasive cervical carcinoma (44.4%) [37]. HCMV infection was also associated with an increased likelihood of HPV16 integration in cervical lesions, a critical step in HSIL progression to cervical cancer [38]. Additionally, co-infection with HPV16 and HCMV was linked to lymph node metastasis in cervical cancer cases [29].

Recent studies have identified high-risk HCMV strains with oncogenic potential, capable of driving cancerous transformations through both onco-modulatory and direct oncogenic mechanisms [35,39,40]. For instance, the high-risk HCMV strain (HCMV-DB) has been shown to inactivate tumor suppressors, activate proto-oncogenes, and upregulate cyclin D1 in human mammary epithelial cells (HMECs). Notably, HMECs transformed by HCMV-DB infection were able to form tumors in mice [40]. These findings suggest a potential role for HCMV in the initiation and progression of various cancers, including cervical cancer [41]. Despite this evidence, the molecular interactions between HCMV and high-risk HPV strains remain unexplored.

This review delves into the potential molecular interactions between HR-HPV and HCMV, emphasizing how viral oncoproteins might collaborate to disrupt cellular functions, evade immune responses, and activate oncogenic pathways. The co-carcinogenesis model proposed here underscores the synergistic effect of these viral infections in driving cervical epithelial transformation and tumor progression.

## 2. Mechanisms of HR-HPV/HCMV-Mediated Cervical Carcinogenesis

Extensive literature and numerous review articles have explored the co-factors associated with HPV in the carcinogenic process, highlighting the complexity and multifactorial nature of HPV-related cancer development. Among these co-factors, viral co-infections, have gained significant attention for their potential role in enhancing HPV-driven oncogenesis. In the following section, it will delve into the molecular mechanisms underlying HR-HPV and HCMV co-carcinogenesis. It will also explore the impact of these viral interactions on key cellular processes and pathways.

### 2.1. HPV Infection Could Facilitate HCMV Entry into Cervical Epithelial Cells

HCMV exploits a variety of cell membrane receptors for binding and entry into host cells, contributing to its broad tissue tropism [42]. The attachment and fusion of HCMV to host cell membranes are mediated by several glycoprotein complexes [43]. The viral entry process relies on the gH/gL protein complex, which assembles with a third glycoprotein, gO to form the gH/gL/gO trimer [44]. Additionally, the gB glycoprotein interacts with gH/gL, facilitating the initial fusion between the virion envelope and the host cell membrane [43]. Another key complex involves gH/gL proteins combined with three viral proteins, UL128, UL130, and UL131, forming the gH/gL/pUL128/pUL130/pUL131 pentamer [45,46]. While the gB and gH/gL/gO complexes are essential for HCMV infection across all cell types, the pentameric complex is specifically required for infection of epithelial cells, enabling viral entry through endocytosis and fusion with endosomal membranes [45,47,48].

In addition, the pentamer complex modulates HCMV tropism by interacting with various cellular receptors, including integrin αvβ3, epidermal growth factor receptor (EGFR), platelet-derived growth factor receptor alpha (PDGFRα), CD147 (Basigin), Ephrin receptor A2 (EphA2), CD90 (Thy-1), Neuropilin-2 (NRP2), and adipocyte plasma membrane-associated protein (APMAP) [43].

#### 2.1.1. Role of EGFR and Integrins

EGFR, in particular, plays a crucial role in HCMV entry, facilitated by its interaction with the viral glycoprotein gB [49]. The activation of the AKT signaling pathway is also necessary for HCMV entry, as demonstrated in EGFR-positive MB468 breast cancer cells, where HCMV entry triggered PI3K pathway activation, a response absent in EGFR-negative MB453 cells unless EGFR was ectopically expressed [50].

HPV proteins, including E5, E6, and E7, have been shown to upregulate EGFR expression in cervical epithelial cells, enhancing EGFR levels on the cell surface [51]. This fact could provide favorable conditions that potentially facilitate HCMV entry. For instance, HPV16 E5 disrupts EGFR ubiquitination, delaying its degradation and increasing receptor availability in keratinocytes [52,53]. Elevated EGFR expression correlates with cervical cancer progression, from LSIL to HSIL and cervical carcinoma [54,55]. Although the key role of EGFR in HCMV entry into epithelial cells is still questionable [56], EGFR activation, alongside integrin αvβ3, appears to be essential for viral infection in epithelial cells [49,57]. Notably, integrin αvβ3 expression is significantly higher in cervical cancer tissues compared to adjacent normal samples and is linked to deeper tumor infiltration [58]. In SiHa cells, αvβ3 associates with the active proteolytic form of matrix metalloproteinase 2 (MMP-2), suggesting a potential role of integrin in regulating matrix degradation [59].

In summary, given the ability of HPV oncoproteins (E5, E6, and E7) to upregulate key cell surface receptors like EGFR and integrin αvβ3, it is plausible that HPV infection may facilitate HCMV entry into epithelial cells. The elevated expression of these receptors, particularly in cervical cancer tissues, suggests a potential co-facilitative role, where HPV-induced receptor upregulation creates a more permissive environment for HCMV infection.

#### 2.1.2. Implications of Co-Receptors and Accessory Proteins

PDGFRα, another receptor linked to HCMV gB binding, shows increased expression in epithelial cells [60,61], including cervical carcinoma lines, particularly those positive for HPV16 [62]. An increased HCMV infection was observed in ARPE-19 epithelial cells expressing PDGFRα, highlighting its role in facilitating viral entry [63]. In addition, the expression of PDGFRα in CERV196 cells (HPV16-positive) was increased compared with HPV-negative SCC cell lines [64]. Although some studies suggest PDGFRα does not directly mediate HCMV entry [63], its activation is known to trigger the PI3K/AKT and focal adhesion kinase (FAK) pathways, which facilitate viral infection [62,65]. These pathways are similarly upregulated by HPV E6/E7 oncoproteins in cervical cancer cells [66,67].

CD90 (Thy-1) and CD147 are additional receptors implicated in HCMV entry into epithelial cells, often showing increased expression in HPV-positive cervical cancer tissues. The evidence indicates that CD90 downregulation impairs HCMV entry [68,69], while CD147 overexpression enhances viral infection [70]. In fact, an overexpression of CD147 has been reported in cervical carcinomas, which correlated with HPV16 E6 expression. These proteins could be potential interactors between these viruses in promoting HCMV infection.

**Figure 1:**
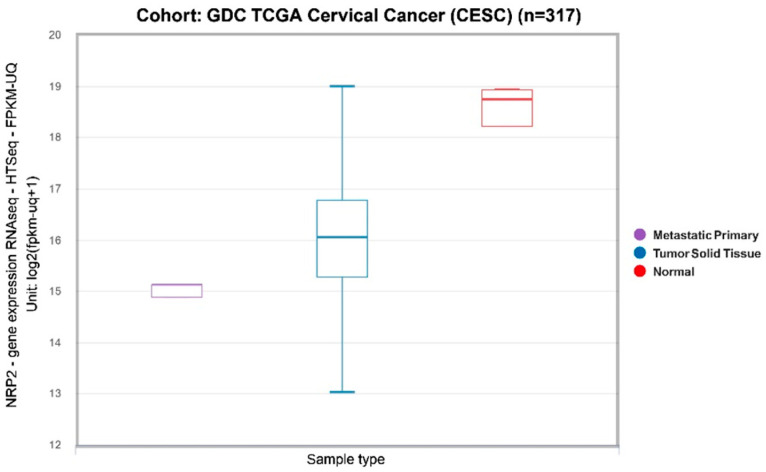
Levels of NRP2 transcripts in primary cervical tumors, metastasis, and normal cervical tissues (GDC TCGA Cervical Cancer, n = 317; *p* = 0.0003, one-way ANOVA). Raw data were extracted from the University of California, Santa Cruz (UCSC) (https://xena.ucsc.edu accessed on 22 July 2024). For the correlation of transcript expression level and phenotypic variables, the UCSC Xena platform for functional genomics data was used (https://xenabrowser.net accessed on 22 July 2024).

Other receptors such as NRP2 and EphA2 also contribute to HCMV entry, with evidence of altered expression in cervical cancer cell lines and tissues. NRP2, in particular, has been associated with HPV E6/E7 expression and may enhance HCMV susceptibility in cervical epithelial cells [71,72]. Conversely, lower NRP2 levels in certain HPV-positive samples suggest variability in receptor expression across different stages of cervical disease [73]. According to the TCGA database, NRP2 expression was higher in normal cervical tissues compared to primary cervical tumors [74], suggesting that normal tissues might be more susceptible to HCMV infection (Figure 1).

Recently, Dong et al. (2023) reported that Ephrin receptor A2 (EphA2) is a functional entry receptor for HCMV in glioblastoma cells [75]. The elevated expression of this molecule in SILs and cervical carcinomas compared to non-tumor cervical epithelium further supports its involvement in viral entry [76]. Notably, ectopic expression of the HPV18 E2 gene in HPV-negative cervical cancer cells significantly increased EphA2 receptor levels [77].

Tetraspanins, among them CD151, were also related to both HPV and HCMV entry into epithelial cells [78]. CD151 is expressed in the basal layer of the squamous cervical epithelium, a common HPV target, and its overexpression has been linked to increased migratory capacity in HeLa cells [79,80]. Depletion of CD151 impairs HCMV infection, underscoring its role in viral entry [81]. Finally, the adipocyte plasma membrane-associated protein (APMAP) was identified as a modulator of HCMV infection in HeLa cells. Specifically, the knockdown of APMAP expression significantly reduced both HCMV entry and IE mRNA expression in these cells [82]. APMAP overexpression is associated with increased invasiveness in HPV-positive cervical cancer cells (HeLa, Caski, and SiHa), highlighting its potential role in facilitating viral entry [83].

Taken together, these findings suggest that HR-HPV may facilitate HCMV entry into cervical epithelial cells by upregulating key receptors involved in this process. However, further functional studies are needed to validate this possibility.

### 2.2. HR-HPV and HCMV Disrupt Key Cellular Processes Involved in Cervical Cancer

HR-HPV contributes significantly to cervical carcinogenesis by exploiting various molecular pathways that regulate cell survival, apoptosis, proliferation, and genomic stability. This section explores the key molecular mechanisms through which HR-HPV and HCMV could cooperate to promote cervical cancer development.

#### 2.2.1. Inhibition of Apoptosis and Cellular Stress Responses

HR-HPV- and HCMV-infected cells exhibit numerous oncogenic properties due to the expression of viral proteins that interfere with cellular processes, notably apoptosis and cellular stress responses [26,84]. For instance, the HPV E5 protein inhibits apoptosis by blocking FasL- and TRAIL-mediated pathways and reducing expression of key endoplasmic reticulum (ER) stress response proteins such as COX-2, XBP-1, and IRE1α [85,86]. Similarly, HCMV-encoded UL38 inhibits apoptosis by targeting the caspase 3-dependent intrinsic pathway and ER-mediated apoptosis after a stress signal [87]. Furthermore, HPV16 E6/E7 upregulates heat shock protein 70 (HSP70) in primary human keratinocytes [88], which helps protect cells from stress-induced apoptosis, thereby disrupting both intrinsic and extrinsic apoptotic pathways [89]. HCMV proteins IE1, IE2, UL36-38, and US3 have also been shown to transactivate the HSP70 promoter in HeLa cells [90]. Although HCMV IE1 and IE2 proteins enhance oncogenic activity in HPV-positive cervical tumor cells by transactivating viral and host genes [90,91], the specific molecular mechanisms of are not fully understood.

Viral proteins further target essential tumor suppressors involved in apoptosis regulation. The HPV E6 protein, in conjunction with E6-AP, targets the tumor suppressor p53 for degradation, thereby preventing apoptosis and interrupting cell cycle arrest [92]. In addition, HCMV vMIA, encoded by the UL37 gene, inhibits mitochondrial membrane permeabilization and cytochrome c release, blocking apoptosis through interactions with the pro-apoptotic protein Bax [93,94]. HPV E6 also degrades other apoptosis-related proteins, including Bak, FADD (Fas-associated protein with death domain), and procaspase-8, further interfering with apoptotic pathways [95,96]. The HCMV UL36 gene encodes another apoptosis inhibitor, vICA, which binds to the caspase-8 prodomain and inhibits Fas-mediated apoptosis in HeLa cells [97]. Furthermore, HCMV IE1 or IE2 expression in HeLa cells confers protection against TNF-α-induced apoptosis independently of Bcl-2 or Bax [98,99].

#### 2.2.2. Modulation of Mitotic Pathways and Cell Immortalization

HR-HPV and HCMV can disrupt normal cell cycle regulation and promote uncontrolled proliferation through various synergistic mechanisms. In HPV-immortalized keratinocytes, tumor necrosis factor-alpha (TNF-α) activates HPV E6/E7 mRNA and cyclin-dependent kinases via a Ras-dependent pathway [100]. HPV E6 and E7 also modulate cell proliferation and survival pathways. E6 activates the mitogen-activated protein kinase (MAPK) and mechanistic target of rapamycin complex 1 (mTORC1) pathways, promoting cellular proliferation [101,102]. Additionally, E7 targets the retinoblastoma protein (RB) for ubiquitination, which releases E2F transcription factors, driving the cell cycle into the S-phase [103,104]. E7 also downregulates p16INK4A (CDKN2A), an essential inhibitor of cyclin-dependent kinases CDK4 and CDK6, while upregulating EGFR and AKT signaling pathways, further enhancing cellular proliferation and oncogenic potential [53,66,105].

Similarly, the HCMV IE1 protein can bind to p107, alleviating p107-mediated transcriptional repression of an E2F-responsive promoter and activating the cyclin E/CDK2 pathway in cervical cancer cells [106,107]. In primary breast epithelial cells, the high-risk HCMV-DB strain has also been shown to activate STAT3, leading to cyclin-D1 upregulation and increased proliferation [40]. Additionally, treatment with HCMV homologue of IL-10 (cmvIL10) has been shown to stimulate cell proliferation via the Janus kinase 1 (JAK1)/STAT3 pathway ([108]).

Beyond promoting uncontrolled proliferation, HR-HPV and HCMV proteins contribute to cellular immortality by activating human telomerase reverse transcriptase (hTERT). This activation sustains telomere length, allowing cells to bypass replicative senescence and continue dividing—a hallmark of cancer. HPV16 E6/E7 has been demonstrated to enhance hTERT promoter activity in HPV-negative C33A cells expressing these oncoproteins [109]. HR-HPV E6/E7 also activates the hTERT promoter in cooperation with the c-Myc oncogene [110,111]. Similarly, the HCMV IE1 protein upregulates hTERT promoter activity in glioblastoma cells, reinforcing its role in cellular immortality [112]. Interestingly, hTERT gene amplification progressively increases from low-grade squamous intraepithelial lesions (LSIL) to high-grade lesions (HSIL) and cervical carcinoma, indicating a link between hTERT expression and cervical lesion progression [113,114].

Collectively, the combined actions of HR-HPV and HCMV proteins underscore their cooperative modulation of proliferative pathways and cell immortalization, which could significantly contribute to the oncogenic transformation of cervical epithelial cells.

#### 2.2.3. Promotion of Angiogenesis

HR-HPV oncoproteins E6 and E7 play a critical role in cervical carcinogenesis by promoting angiogenesis. In human keratinocytes, HPV16 E6/E7 expression reduces the levels of angiogenesis inhibitors thrombospondin-1 and maspin, while increasing levels of vascular endothelial growth factor (VEGF), a key driver of angiogenesis. VEGF promotes new blood vessel formation, supplying nutrients and oxygen to tumors and facilitating their growth and survival [115]. Additionally, Wang et al. demonstrated that HPV E7 upregulates the ribonucleotide reductase regulatory subunit M2 (RRM2) in cervical cancer cells, thereby inducing angiogenesis via the ROS-ERK1/2-HIF-1α-VEGF pathway [116]. Similarly, the HCMV secretome contains numerous angiogenic proteins [117]. Treatment of endothelial cells (HUVEC) with the HCMV secretome has been shown to stimulate vessel formation [118]. Moreover, the HCMV-encoded chemokine receptor US28 enhances COX-2 expression through NF-κB activation, leading to increased VEGF production [119].

#### 2.2.4. Tumorigenicity and Induction of Epithelial-to-Mesenchymal Transition (EMT)

HR-HPV oncoproteins E6 and E7 play a pivotal role in promoting the malignant progression of cervical cancer by increasing tumorigenicity and inducing EMT [120]. In a loss-of-function study, Yoshinouchi et al. (2003) demonstrated that HPV16 E6 induces anchorage-independent growth in cervical cancer cells and promotes tumor formation in NOD/SCID mice [121]. Similarly, silencing HPV18 E6 or E7 with siRNA inhibited colony formation in HeLa cervical cancer cells [122], while the trasfection of normal cervical cells with HPV16 E6/E7 promoted colony formation [123]. Likewise, HCMV IE2 has been shown to significantly enhance anchorage-independent growth in HeLa cells, a trait associated with high transformation and tumorigenic potential [91]. Interestingly, the integration of HCMV DNA into the host genome may not directly impact HPV16 transcript expression [124]. Thus, the cooperative interaction between HPV and HCMV proteins may synergistically drive cellular transformation and tumorigenesis. Indeed, one study reported that co-infection with HCMV IE and HPV16 was sufficient to transform human cervical epithelial cells and induce tumor formation in nude mice [125].

EMT is a cellular process that enables epithelial cells to acquire mesenchymal characteristics, such as increased motility, invasiveness, and resistance to apoptosis [126]. In cervical cancer cells, the HPV16 E2, E6, and E7 oncoproteins were found to upregulate vimentin and Twist2 expression while downregulating E-cadherin [127]. Additionally, HPV16 E7 has been shown to increase fibronectin levels in normal human epithelial cells [128], and ectopic expression of HPV16 E6/E7 in HPV-negative cells led to elevated levels of α-smooth muscle actin (α-SMA) and vimentin proteins [120]. By activating EMT, the E6 and E7 oncoproteins contribute to the aggressive behavior of cervical cancer cells, facilitating their invasion into surrounding tissues and their potential metastasis to distant sites [128]. Similarly, tumor samples from mice inoculated with HCMV-infected cells revealed decreased E-cadherin expression and increased vimentin, indicating EMT induction [40]. In addition, a reduction in epithelial cell adhesion molecule (EpCAM) expression was observed in human mammary epithelial cells infected with HCMV [129].

Based on these findings, it is hypothesized that HR-HPV and HCMV proteins could work together to inhibit apoptosis and cellular stress responses, disrupt cell cycle regulation, and promote cell immortalization, angiogenesis, and EMT—key processes that fuel cancer progression. This cooperation may support the integration of HPV DNA into the host genome, thereby potentially contributing to cervical carcinogenesis.

### 2.3. Immune Suppression Induced by HR-HPV and HCMV Could Facilitate Cervical Lesions Progression

HR-HPV and HCMV utilize complex strategies to evade and suppress immune responses, targeting key processes such as innate immune recognition, cytokine signaling, and antigen presentation [130,131]. These immune evasion tactics enable viral persistence within tissues, contributing to a microenvironment conducive to cancer progression. The following sections will explore how HR-HPV and HCMV disrupt these critical immunological pathways, potentially cooperating in the development of cervical cancer.

#### 2.3.1. Evasion of Innate Immune Response

The innate immune system serves as the first line of defense against viral infections, primarily through the activation of Toll-like receptors (TLRs) that recognize pathogen-associated molecular patterns (PAMPs) [132]. TLRs trigger signaling cascades that lead to the production of interferons (IFNs) and pro-inflammatory cytokines [133], which are critical for antiviral defense. However, primary HR-HPV infection in cervical epithelium creates a favorable environment for HCMV secondary infection, and HCMV immune evasion strategies may help HR-HPV-infected cells evade immune surveillance [134,135]. In HPV16-positive cervical cancer cells, TLR9, which recognizes double-stranded DNA, is downregulated due to HPV16 E6/E7 expression, similar to the effects seen with HPV38 E6/E7 proteins [136,137]. In contrast, increased expression of TLRs, including TLR3, TLR7, TLR8, TLR9, and TLR2, has been associated with HPV16 clearance or control in cervical cancer tissues, highlighting the role of TLRs in defending against HPV infection [138]. This fact suggests that TLRs play pivotal roles in the host immune defense against HPV-infected cells. HCMV-encoded proteins, such as US7 and US8, degrade TLR3 and TLR4, while HCMV miR-UL112-3p targets and inhibits TLR2, modulating the TLR2/IRAK1/NFκB signaling pathway [139,140]. Interestingly, Harwani et al. reported that TLR2, TLR3, TLR4, and TLR9 are able to inhibit HCMV infection in ectocervical explants through the production of IFN-β [141], which is a target of HPV16 E6 oncoprotein [142].

In summary, both HR-HPV and HCMV interfere with TLR-mediated innate immune responses, contributing to the evasion of antiviral defenses. These disruptions could enhance viral persistence and facilitate the progression of cervical lesions.

#### 2.3.2. Interference with NF-κB Signaling

The NF-κB signaling pathway plays a pivotal role in the host immune response by regulating the expression of IFNs and pro-inflammatory cytokines in response to infections. This pathway is crucial for mounting an effective antiviral response, promoting inflammation, and activating immune cells [143].

HPV16 E7 disrupts NF-κB signaling by preventing the nuclear translocation of NF-κB p65, interacting with the IKK complex, and impairing IκBα phosphorylation, thereby inhibiting NF-κB activation [144,145]. HCMV miR-US5-1 and miR-UL112-3p further suppress NF-κB signaling by targeting IKKα and IKKβ, reducing pro-inflammatory cytokines IL-6 and CCL5 after TNF-α stimulation [146]. Additionally, HCMV UL26 blocks IKK phosphorylation, and HPV16 E6 and E7 disrupt NF-κB transcriptional activity by increasing p105 (NFKB1) and p100 (NFKB2) levels, and inhibiting the coactivator functions of CBP/p300 [147,148,149,150]. Overall, these data suggest that HR-HPV and HCMV may synergistically inhibit NF-κB signaling pathways, suppressing the production of pro-inflammatory cytokines and weakening the immune response against infected cells, thus promoting viral persistence.

#### 2.3.3. Suppression of Interferon Responses

Interferons (IFNs) are key antiviral cytokines that play an essential role in controlling viral infections. Type I IFNs, such as IFN-α and IFN-β, initiate antiviral defenses by activating interferon regulatory factors (IRFs) and stimulating the expression of interferon-stimulated genes (ISGs) [151]. HR-HPV and HCMV have developed strategies to suppress IFN signaling, allowing them to evade the host’s antiviral immune response and enhance their survival within the host.

HR-HPV disrupts type I IFN signaling to evade the antiviral immune response. For instance, HPV16 E6 binds to IRF3, reducing IFN-β expression, while HPV16 E5 modulates IRF3 and IRF7, suppressing stromal type I IFNs [142,152]. HR-HPVs also downregulate ISGs, disrupting the production of IFN-β and IFN-λ, thereby impairing the host’s antiviral responses [153]. Similarly, HCMV proteins, such as UL23, help to evade IFN-γ-mediated immune responses by suppressing ISG expression, further facilitating viral persistence in infected cells [154]. Consequently, HR-HPV and HCMV can jointly disrupt the interferon response, allowing for viral persistence and potentially contributing to the progression of cervical lesions.

#### 2.3.4. Induction of Immunosuppressive Cytokines

Cytokines are signaling molecules that mediate and regulate immunity, inflammation, and hematopoiesis [151]. By upregulating these cytokines, the viruses inhibit immune cell activation and promote an immunosuppressive microenvironment, which favors viral persistence and tumor progression. HR-HPV and HCMV exploit the immunosuppressive properties of certain cytokines, to create an environment that dampens immune responses. For instance, it was reported that HPV16 E2 can upregulate the expression of IL-10 in cervical cancer cells, contributing to local immune suppression and aiding viral persistence [155,156,157]. Interestingly, the HCMV UL111A gene encodes cmvIL-10, a viral homolog of human IL-10, which promotes monocyte conversion to an immunosuppressive phenotype and activates STAT3 in cervical cancer cells [158,159]. Moreover, HCMV miR-US5-1 and miR-UL112-3p target the IKKα and IKKβ components of the NF-κB signaling pathway, reducing the transcript levels of IL-6 and CCL5 pro-inflammatory cytokines after the stimulation of HeLa cells with IL-1β or TNF-α [146]. HCMV also encodes some G-protein coupled receptors (GPCRs) [160]. Among them, HCMV US28 is able to bind and sequester cellular chemokines as a mechanism to evade the host immune response [161,162]. These facts suggest that local immunosuppression induced by HCMV proteins could potentially facilitate the proliferation of HPV-positive cells.

The transforming growth factor-β1 (TGF-β1) is another important molecule related to HR-HPV persistence and cervical cancer development [163]. TGF-β1 reduced the levels of both interleukin-2 (IL-2) and IFN-γ, impairing the function of natural killer (NK) cells and cytotoxic T lymphocytes [164]. The overexpression of TGF-β1 mRNA was associated with the progression from LSIL to HSIL [165] and cervical carcinomas [164]. Moreover, the expression of TGF-β1 correlated with HPV infection in cervical adenocarcinoma [166] as well as with HPV E7 in cervical abnormalities [164]. In line with this, it was reported that HPV E6 and E7 increase the TGF-β1 promoter activity in cervical cancer cells [167]. HCMV infection also induces TGF-β1 production in different cancer cell types [168,169]. Furthermore, it was shown that the HCMV IE2 protein regulates the transcription of the TGF-β1 gene interacting with the Egr-1 DNA-binding protein in glioblastoma cells [170]. The increased production of TGF-β1 HCMV inhibits GM-CSF expression in HCMV-infected Saos-2 cells [169].

Overall, these data indicate that HR-HPV and HCMV contribute to an immunosuppressive microenvironment by inducing cytokines such as IL-10 and TGF-β1. This immune-suppressive environment could enable infected cells to evade immune detection and promotes the progression of cervical cancer.

#### 2.3.5. Disruption of Antigen Presentation

The presentation of viral antigens by major histocompatibility complex (MHC) molecules is essential for the activation of T cells, which are critical for the recognition and elimination of infected cells. HR-HPV and HCMV manipulate antigen presentation pathways by downregulating MHC class I and II molecules. For instance, HPV16 E5 and E6 downregulate HLA-I, reducing recognition by CD8+ T cells and suppressing the antigen presentation pathways essential for effective immune clearance [171,172]. Likewise, HCMV US11 also targets MHC-I, impairing immune recognition and facilitating persistence [173,174]. Moreover, HCMV US28 inhibits MHC-II, evading the immune recognition of HCMV antigens by specific CD4+ T cells [174]. Therefore, HR-HPV and HCMV could hinder antigen presentation pathways, potentially reducing the host’s ability to elicit an effective T-cell-mediated immune response, thereby promoting viral persistence and cervical lesion progression.

**Figure 2:**
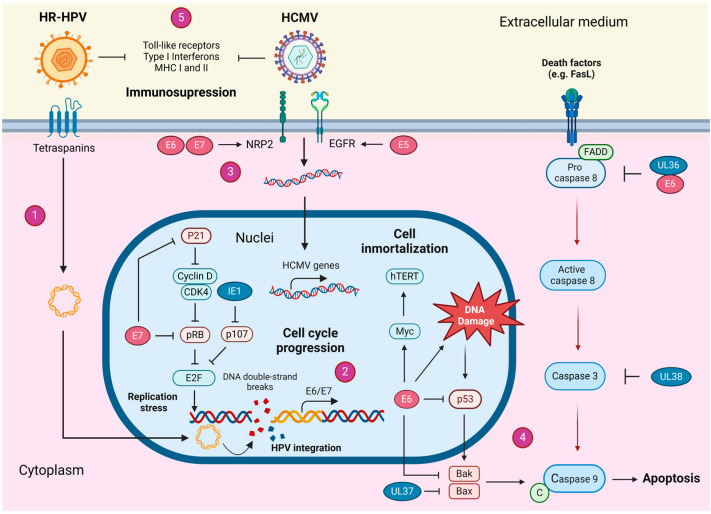
A proposed model of HR-HPV and HCMV cooperation for cervical cancer development. 1: HR-HPV infects cervical epithelial cells and promotes DNA damage and double-strand breaks, which favors the viral integration into host genome; 2: the constitutive expression of E6 and E7 oncoproteins inactivate p53 and pRB tumor suppressors, leading to cell cycle progression, replication stress and genomic instability; 3: HR-HPV oncoproteins increase the expression of cellular receptors such as EGFR and NRP2, facilitating HCMV cell entry; 4: the expression of HCMV UL36-38 proteins protect HPV-infected cells from DNA damage-induced apoptosis; 5: the expression of HR-HPV and HCMV proteins inhibits key mediators of the antiviral immune response (e.g., secretion of type I interferons), inducing immunosuppression. Brown shapes with white text represent HR-HPV oncoproteins. Blue shapes with white text indicate HCMV proteins. Created by BioRender.com.

Taken together, clinical and experimental evidence indicates that HCMV may contribute to the initiation and progression of HPV-positive cervical tumors by facilitating immune evasion. A hypothetical model depicting the cooperative roles of HR-HPV and HCMV proteins in cervical cancer development is illustrated in Figure 2.

## 3. Conclusions

A single infection with HR-HPV is insufficient to drive cervical carcinogenesis, indicating that additional co-factors contribute to disease progression. Although the oncogenic potential of HCMV remains a subject of debate, certain HCMV strains, particularly high-risk variants, have shown the ability to activate oncogenic pathways that promote epithelial cell transformation. The co-presence of HR-HPV and HCMV has been observed in cervical lesions, with a higher prevalence noted from LSIL to cervical squamous carcinoma. However, it is critical to distinguish between low- and high-risk oncogenic HCMV strains in cervical epithelial infections to better identify patients at increased risk for lesion progression.

While the exact molecular mechanisms underlying HR-HPV and HCMV cooperation are not fully understood, this review highlights several potential interactions. Firstly, HR-HPV may facilitate HCMV entry into cervical epithelial cells by upregulating host cell receptors that are crucial for viral attachment and fusion. Secondly, HR-HPV and HCMV may synergistically contribute to cervical carcinogenesis, with viral proteins disrupting key cellular processes involved in cervical cancer. Thirdly, both HR-HPV and HCMV modulate the local immune environment, allowing infected cells to evade host immune surveillance and promoting lesion persistence.

To validate these hypotheses and further elucidate the cooperative mechanisms between HR-HPV and HCMV in cervical cancer development, additional in vitro and in vivo studies are needed. Understanding these interactions may provide valuable insights into the pathogenesis of cervical cancer and identify novel therapeutic targets.

## Data Availability

All datasets used in this study are available from UCSC Xena browser (https://xenabrowser.net/datapages/?cohort GDC TCGA Cervical Cancer (CESC) (accessed on 22 July 2024)).

## References

[1] Bray F., Laversanne M., Sung H., Ferlay J., Siegel R.L., Soerjomataram I., Jemal A. (2024). Global cancer statistics 2022: GLOBOCAN estimates of incidence and mortality worldwide for 36 cancers in 185 countries. CA Cancer J. Clin..

[2] Yang M., Du J., Lu H., Xiang F., Mei H., Xiao H. (2022). Global trends and age-specific incidence and mortality of cervical cancer from 1990 to 2019: An international comparative study based on the Global Burden of Disease. BMJ Open.

[3] Falcaro M., Castañon A., Ndlela B., Checchi M., Soldan K., Lopez-Bernal J., Elliss-Brookes L., Sasieni P. (2021). The effects of the national HPV vaccination programme in England, UK, on cervical cancer and grade 3 cervical intraepithelial neoplasia incidence: A register-based observational study. Lancet.

[4] Palmer T.J., Kavanagh K., Cuschieri K., Cameron R., Graham C., Wilson A., Roy K. (2024). Invasive cervical cancer incidence following bivalent human papillomavirus vaccination: A population-based observational study of age at immunization, dose, and deprivation. J. Natl. Cancer Inst..

[5] zur Hausen H. (2009). Papillomaviruses in the causation of human cancers—A brief historical account. Virology.

[6] Okunade K.S. (2020). Human papillomavirus and cervical cancer. J. Obstet. Gynaecol..

[7] Thompson A.B., Flowers L.C., Hussen S.A. (2020). Human Papillomavirus (HPV). Sexually Transmitted Infections in Adolescence and Young Adulthood: A Practical Guide for Clinicians.

[8] Demarco M., Hyun N., Carter-Pokras O., Raine-Bennett T.R., Cheung L., Chen X., Hammer A., Campos N., Kinney W., Gage J.C. (2020). A study of type-specific HPV natural history and implications for contemporary cervical cancer screening programs. EClinicalMedicine.

[9] Bowden S.J., Kyrgiou M. (2020). Human papillomavirus. Obstet. Gynaecol. Reprod. Med..

[10] Nelson C.W., Mirabello L. (2023). Human papillomavirus genomics: Understanding carcinogenicity. Tumour Virus Res..

[11] Blanco R., Carrillo-Beltrán D., Muñoz J.P., Corvalán A.H., Calaf G.M., Aguayo F. (2021). Human Papillomavirus in Breast Carcinogenesis: A Passenger, a Cofactor, or a Causal Agent?. Biology.

[12] Aguayo F., Muñoz J.P., Perez-Dominguez F., Carrillo-Beltrán D., Oliva C., Calaf G.M., Blanco R., Nuñez-Acurio D. (2020). High-Risk Human Papillomavirus and Tobacco Smoke Interactions in Epithelial Carcinogenesis. Cancers.

[13] Hildesheim A., Herrero R., Castle P.E., Wacholder S., Bratti M.C., Sherman M.E., Lorincz A.T., Burk R.D., Morales J., Rodriguez A.C. (2001). HPV co-factors related to the development of cervical cancer: Results from a population-based study in Costa Rica. Br. J. Cancer.

[14] Luhn P., Walker J., Schiffman M., Zuna R.E., Dunn S.T., Gold M.A., Smith K., Mathews C., Allen R.A., Zhang R. (2013). The role of co-factors in the progression from human papillomavirus infection to cervical cancer. Gynecol. Oncol..

[15] Appleby P., Beral V., de González A.B., Colin D., Franceschi S., Goodhill A., Green J., Peto J., Plummer M., International Collaboration of Epidemiological Studies of Cervical Cancer (2007). Cervical cancer and hormonal contraceptives: Collaborative reanalysis of individual data for 16 573 women with cervical cancer and 35 509 women without cervical cancer from 24 epidemiological studies. Lancet.

[16] Imajoh M., Hashida Y., Nemoto Y., Oguri H., Maeda N., Furihata M., Fukaya T., Daibata M. (2012). Detection of Merkel cell polyomavirus in cervical squamous cell carcinomas and adenocarcinomas from Japanese patients. Virol. J..

[17] Sausen D.G., Shechter O., Gallo E.S., Dahari H., Borenstein R. (2023). Herpes Simplex Virus, Human Papillomavirus, and Cervical Cancer: Overview, Relationship, and Treatment Implications. Cancers.

[18] Chavoshpour-Mamaghani S., Shoja Z., Jalilvand S. (2024). The Prevalence of Epstein-Barr Virus in Normal, Premalignant, and Malignant Uterine Cervical Samples in Iran. Intervirology.

[19] Akbari E., Milani A., Seyedinkhorasani M., Bolhassani A. (2023). HPV co-infections with other pathogens in cancer development: A comprehensive review. J. Med. Virol..

[20] Li R., Meng W., Zuo Y., Xu Y., Wu S. (2024). The cervical cancer related distribution, coinfection and risk of 15 HPV types in Baoan, Shenzhen, in 2017–2023. Virol. J..

[21] Blanco R., Carrillo-Beltrán D., Osorio J.C., Calaf G.M., Aguayo F. (2020). Role of Epstein-Barr Virus and Human Papillomavirus Coinfection in Cervical Cancer: Epidemiology, Mechanisms and Perspectives. Pathogens.

[22] Daxnerova Z., Berkova Z., Kaufman R.H., Adam E. (2003). Detection of Human Cytomegalovirus DNA in 986 Women Studied for Human Papillomavirus-Associated Cervical Neoplasia. J. Low. Genit. Tract Dis..

[23] de Lima M.A.P., Neto P.J.N., Lima L.P.M., Gonçalves Júnior J., Teixeira Junior A.G., Teodoro I.P.P., Facundo H.T., da Silva C.G.L., Lima M.V.A. (2018). Association between Epstein-Barr virus (EBV) and cervical carcinoma: A meta-analysis. Gynecol. Oncol..

[24] Li S., Wen X. (2017). Seropositivity to herpes simplex virus type 2, but not type 1 is associated with cervical cancer: NHANES (1999-2014). BMC Cancer.

[25] Santos C.A.Q. (2016). Cytomegalovirus and Other β-Herpesviruses. Semin. Nephrol..

[26] Yu C., He S., Zhu W., Ru P., Ge X., Govindasamy K. (2023). Human cytomegalovirus in cancer: The mechanism of HCMV-induced carcinogenesis and its therapeutic potential. Front. Cell. Infect. Microbiol..

[27] Michaelis M., Doerr H.W., Cinatl J. (2009). The Story of Human Cytomegalovirus and Cancer: Increasing Evidence and Open Questions. Neoplasia.

[28] Herbein G. (2024). Cellular Transformation by Human Cytomegalovirus. Cancers.

[29] Chen T.-M., Chang C.-F., Chen Y.-H., Chen C.-A., Wu C.-C., Hsieh C.-Y. (1996). Coexistence of human cytomegalovirus and human papillomavirus type 16 correlates with lymph node metastasis in cervical cancer. J. Cancer Res. Clin. Oncol..

[30] Richardson A.K., Currie M.J., Robinson B.A., Morrin H., Phung Y., Pearson J.F., Anderson T.P., Potter J.D., Walker L.C. (2015). Cytomegalovirus and Epstein-Barr virus in breast cancer. PLoS ONE.

[31] Pacsa A.S., Kummerländer L., Pejtsik B., Pali K. (1975). Herpesvirus antibodies and antigens in patients with cervical anaplasia and in controls. J. Natl. Cancer Inst..

[32] Cobbs C.S., Harkins L., Samanta M., Gillespie G.Y., Bharara S., King P.H., Nabors L.B., Cobbs C.G., Britt W.J. (2002). Human cytomegalovirus infection and expression in human malignant glioma. Cancer Res..

[33] Giraldo G., Beth E., Huang E.S. (1980). Kaposi’s sarcoma and its relationship to cytomegalovirus (CMNV). III. CMV DNA and CMV early antigens in Kaposi’s sarcoma. Int. J. Cancer.

[34] Bai B., Wang X., Chen E., Zhu H. (2016). Human cytomegalovirus infection and colorectal cancer risk: A meta-analysis. Oncotarget.

[35] Herbein G. (2022). High-Risk Oncogenic Human Cytomegalovirus. Viruses.

[36] Cobbs C. (2019). Cytomegalovirus is a tumor-associated virus: Armed and dangerous. Curr. Opin. Virol..

[37] Marinho-Dias J., Sousa H. (2013). Cytomegalovirus infection and cervical cancer: From past doubts to present questions. Acta Med. Port..

[38] Szostek S., Zawilinska B., Kopec J., Kosz-Vnenchak M. (2009). Herpesviruses as possible cofactors in HPV-16-related oncogenesis. Acta Biochim. Pol..

[39] El Baba R., Pasquereau S., Haidar Ahmad S., Diab-Assaf M., Herbein G. (2022). Oncogenic and Stemness Signatures of the High-Risk HCMV Strains in Breast Cancer Progression. Cancers.

[40] Kumar A., Tripathy M.K., Pasquereau S., Al Moussawi F., Abbas W., Coquard L., Khan K.A., Russo L., Algros M.P., Valmary-Degano S. (2018). The Human Cytomegalovirus Strain DB Activates Oncogenic Pathways in Mammary Epithelial Cells. EBioMedicine.

[41] Herbein G. (2018). The Human Cytomegalovirus, from Oncomodulation to Oncogenesis. Viruses.

[42] Gerna G., Kabanova A., Lilleri D. (2019). Human Cytomegalovirus Cell Tropism and Host Cell Receptors. Vaccines.

[43] Nguyen C.C., Kamil J.P. (2018). Pathogen at the Gates: Human Cytomegalovirus Entry and Cell Tropism. Viruses.

[44] Heldwein E.E. (2016). gH/gL supercomplexes at early stages of herpesvirus entry. Curr. Opin. Virol..

[45] Wang D., Shenk T. (2005). Human cytomegalovirus UL131 open reading frame is required for epithelial cell tropism. J. Virol..

[46] Wang D., Shenk T. (2005). Human cytomegalovirus virion protein complex required for epithelial and endothelial cell tropism. Proc. Natl. Acad. Sci. USA.

[47] Ryckman B.J., Jarvis M.A., Drummond D.D., Nelson J.A., Johnson D.C. (2006). Human cytomegalovirus entry into epithelial and endothelial cells depends on genes UL128 to UL150 and occurs by endocytosis and low-pH fusion. J. Virol..

[48] Zhou M., Lanchy J.M., Ryckman B.J. (2015). Human Cytomegalovirus gH/gL/gO Promotes the Fusion Step of Entry into All Cell Types, whereas gH/gL/UL128-131 Broadens Virus Tropism through a Distinct Mechanism. J. Virol..

[49] Wang X., Huong S.M., Chiu M.L., Raab-Traub N., Huang E.S. (2003). Epidermal growth factor receptor is a cellular receptor for human cytomegalovirus. Nature.

[50] Ee X., Meraner P., Lu P., Perreira J.M., Aker A.M., McDougall W.M., Zhuge R., Chan G.C., Gerstein R.M., Caposio P. (2019). OR14I1 is a receptor for the human cytomegalovirus pentameric complex and defines viral epithelial cell tropism. Proc. Natl. Acad. Sci. USA.

[51] Wasson C.W., Morgan E.L., Muller M., Ross R.L., Hartley M., Roberts S., Macdonald A. (2017). Human papillomavirus type 18 E5 oncogene supports cell cycle progression and impairs epithelial differentiation by modulating growth factor receptor signalling during the virus life cycle. Oncotarget.

[52] Zhang B., Srirangam A., Potter D.A., Roman A. (2005). HPV16 E5 protein disrupts the c-Cbl-EGFR interaction and EGFR ubiquitination in human foreskin keratinocytes. Oncogene.

[53] Akerman G.S., Tolleson W.H., Brown K.L., Zyzak L.L., Mourateva E., Engin T.S., Basaraba A., Coker A.L., Creek K.E., Pirisi L. (2001). Human papillomavirus type 16 E6 and E7 cooperate to increase epidermal growth factor receptor (EGFR) mRNA levels, overcoming mechanisms by which excessive EGFR signaling shortens the life span of normal human keratinocytes. Cancer Res..

[54] Boiko I.V., Mitchell M.F., Hu W., Pandey D.K., Mathevet P., Malpica A., Hittelman W.N. (1998). Epidermal growth factor receptor expression in cervical intraepithelial neoplasia and its modulation during an alpha-difluoromethylornithine chemoprevention trial. Clin. Cancer Res..

[55] Fukazawa E.M., Baiocchi G., Soares F.A., Kumagai L.Y., Faloppa C.C., Badiglian-Filho L., Coelho F.R., Goncalves W.J., Costa R.L., Goes J.C. (2014). Cox-2, EGFR, and ERBB-2 expression in cervical intraepithelial neoplasia and cervical cancer using an automated imaging system. Int. J. Gynecol. Pathol..

[56] Isaacson M.K., Feire A.L., Compton T. (2007). Epidermal growth factor receptor is not required for human cytomegalovirus entry or signaling. J. Virol..

[57] Wang X., Huang D.Y., Huong S.M., Huang E.S. (2005). Integrin alphavbeta3 is a coreceptor for human cytomegalovirus. Nat. Med..

[58] Feng X., Li J. (2020). Relationship between integrin αvβ3 and αvβ1 expression levels and clinicopathological characteristics of cervical cancer. Trop. J. Pharm. Res..

[59] Chattopadhyay N., Mitra A., Frei E., Chatterjee A. (2001). Human cervical tumor cell (SiHa) surface alphavbeta3 integrin receptor has associated matrix metalloproteinase (MMP-2) activity. J. Cancer Res. Clin. Oncol..

[60] Yang Z., Tang X., McMullen T.P.W., Brindley D.N., Hemmings D.G. (2021). PDGFRalpha Enhanced Infection of Breast Cancer Cells with Human Cytomegalovirus but Infection of Fibroblasts Increased Prometastatic Inflammation Involving Lysophosphatidate Signaling. Int. J. Mol. Sci..

[61] Taja-Chayeb L., Chavez-Blanco A., Martinez-Tlahuel J., Gonzalez-Fierro A., Candelaria M., Chanona-Vilchis J., Robles E., Duenas-Gonzalez A. (2006). Expression of platelet derived growth factor family members and the potential role of imatinib mesylate for cervical cancer. Cancer Cell Int..

[62] Soroceanu L., Akhavan A., Cobbs C.S. (2008). Platelet-derived growth factor-alpha receptor activation is required for human cytomegalovirus infection. Nature.

[63] Vanarsdall A.L., Wisner T.W., Lei H., Kazlauskas A., Johnson D.C. (2012). PDGF receptor-alpha does not promote HCMV entry into epithelial and endothelial cells but increased quantities stimulate entry by an abnormal pathway. PLoS Pathog..

[64] Aderhold C., Umbreit C., Faber A., Sauter A., Sommer J.U., Birk R., Erben P., Hofheinz R.D., Stern-Straeter J., Hormann K. (2013). Chemotherapeutic alteration of VEGF, PDGF and PDGFRalpha/beta expression under 5-FU vs. docetaxel in HPV-transformed squamous cell carcinoma compared to HPV-negative HNSCC in vitro. Anticancer Res..

[65] Feire A.L., Koss H., Compton T. (2004). Cellular integrins function as entry receptors for human cytomegalovirus via a highly conserved disintegrin-like domain. Proc. Natl. Acad. Sci. USA.

[66] Menges C.W., Baglia L.A., Lapoint R., McCance D.J. (2006). Human papillomavirus type 16 E7 up-regulates AKT activity through the retinoblastoma protein. Cancer Res..

[67] McCormack S.J., Brazinski S.E., Moore J.L., Werness B.A., Goldstein D.J. (1997). Activation of the focal adhesion kinase signal transduction pathway in cervical carcinoma cell lines and human genital epithelial cells immortalized with human papillomavirus type 18. Oncogene.

[68] Li Q., Wilkie A.R., Weller M., Liu X., Cohen J.I. (2015). THY-1 Cell Surface Antigen (CD90) Has an Important Role in the Initial Stage of Human Cytomegalovirus Infection. PLoS Pathog..

[69] Vanarsdall A.L., Pritchard S.R., Wisner T.W., Liu J., Jardetzky T.S., Johnson D.C. (2018). CD147 Promotes Entry of Pentamer-Expressing Human Cytomegalovirus into Epithelial and Endothelial Cells. mBio.

[70] Zhao Y., Luan J., Feng F., Chen Z.N. (2017). CD147 and HPV16 oncoprotein expression in cervical squamous cell carcinoma and the clinical implications. Int. J. Clin. Exp. Pathol..

[71] Martinez-Martin N., Marcandalli J., Huang C.S., Arthur C.P., Perotti M., Foglierini M., Ho H., Dosey A.M., Shriver S., Payandeh J. (2018). An Unbiased Screen for Human Cytomegalovirus Identifies Neuropilin-2 as a Central Viral Receptor. Cell.

[72] Fujii T., Shimada K., Asano A., Tatsumi Y., Yamaguchi N., Yamazaki M., Konishi N. (2016). MicroRNA-331-3p Suppresses Cervical Cancer Cell Proliferation and E6/E7 Expression by Targeting NRP2. Int. J. Mol. Sci..

[73] Zhang M., Song Y., Zhai F. (2018). ARFHPV E7 oncogene, lncRNA HOTAIR, miR-331-3p and its target, NRP2, form a negative feedback loop to regulate the apoptosis in the tumorigenesis in HPV positive cervical cancer. J. Cell. Biochem..

[74] Goldman M.J., Craft B., Hastie M., Repecka K., McDade F., Kamath A., Banerjee A., Luo Y., Rogers D., Brooks A.N. (2020). Visualizing and interpreting cancer genomics data via the Xena platform. Nat. Biotechnol..

[75] Dong X.D., Li Y., Li Y., Sun C., Liu S.X., Duan H., Cui R., Zhong Q., Mou Y.G., Wen L. (2023). EphA2 is a functional entry receptor for HCMV infection of glioblastoma cells. PLoS Pathog..

[76] Huang C., Chen Z., He Y., He Z., Ban Z., Zhu Y., Ding L., Yang C., Jeong J.H., Yuan W. (2021). EphA2 promotes tumorigenicity of cervical cancer by up-regulating CDK6. J. Cell. Mol. Med..

[77] Fuentes-Gonzalez A.M., Munoz-Bello J.O., Manzo-Merino J., Contreras-Paredes A., Pedroza-Torres A., Fernandez-Retana J., Perez-Plasencia C., Lizano M. (2019). Intratype variants of the E2 protein from human papillomavirus type 18 induce different gene expression profiles associated with apoptosis and cell proliferation. Arch. Virol..

[78] Fast L.A., Mikulicic S., Fritzen A., Schwickert J., Boukhallouk F., Hochdorfer D., Sinzger C., Suarez H., Monk P.N., Yanez-Mo M. (2018). Inhibition of Tetraspanin Functions Impairs Human Papillomavirus and Cytomegalovirus Infections. Int. J. Mol. Sci..

[79] Scheffer K.D., Gawlitza A., Spoden G.A., Zhang X.A., Lambert C., Berditchevski F., Florin L. (2013). Tetraspanin CD151 mediates papillomavirus type 16 endocytosis. J. Virol..

[80] Testa J.E., Brooks P.C., Lin J.M., Quigley J.P. (1999). Eukaryotic expression cloning with an antimetastatic monoclonal antibody identifies a tetraspanin (PETA-3/CD151) as an effector of human tumor cell migration and metastasis. Cancer Res..

[81] Hochdorfer D., Florin L., Sinzger C., Lieber D. (2016). Tetraspanin CD151 Promotes Initial Events in Human Cytomegalovirus Infection. J. Virol..

[82] Ye X., Gui X., Freed D.C., Ku Z., Li L., Chen Y., Xiong W., Fan X., Su H., He X. (2019). Identification of adipocyte plasma membrane-associated protein as a novel modulator of human cytomegalovirus infection. PLoS Pathog..

[83] Zhu X., Xiang Z., Zou L., Chen X., Peng X., Xu D. (2021). APMAP Promotes Epithelial-Mesenchymal Transition and Metastasis of Cervical Cancer Cells by Activating the Wnt/beta-catenin Pathway. J. Cancer.

[84] Rosendo-Chalma P., Antonio-Vejar V., Ortiz Tejedor J.G., Ortiz Segarra J., Vega Crespo B., Bigoni-Ordonez G.D. (2024). The Hallmarks of Cervical Cancer: Molecular Mechanisms Induced by Human Papillomavirus. Biology.

[85] Kabsch K., Alonso A. (2002). The human papillomavirus type 16 E5 protein impairs TRAIL- and FasL-mediated apoptosis in HaCaT cells by different mechanisms. J. Virol..

[86] Sudarshan S.R., Schlegel R., Liu X. (2010). The HPV-16 E5 protein represses expression of stress pathway genes XBP-1 and COX-2 in genital keratinocytes. Biochem. Biophys. Res. Commun..

[87] Terhune S., Torigoi E., Moorman N., Silva M., Qian Z., Shenk T., Yu D. (2007). Human cytomegalovirus UL38 protein blocks apoptosis. J. Virol..

[88] Liao W.J., Fan P.S., Fu M., Fan X.L., Liu Y.F. (2005). Increased expression of 70 kD heat shock protein in cultured primary human keratinocytes induced by human papillomavirus 16 E6/E7 gene. Chin. Med. J..

[89] Albakova Z., Armeev G.A., Kanevskiy L.M., Kovalenko E.I., Sapozhnikov A.M. (2020). HSP70 Multi-Functionality in Cancer. Cells.

[90] Colberg-Poley A.M., Santomenna L.D., Harlow P.P., Benfield P.A., Tenney D.J. (1992). Human cytomegalovirus US3 and UL36-38 immediate-early proteins regulate gene expression. J. Virol..

[91] Yuo C.Y., Wu G.J., Huang E.S., Wu F.Y., Wu C.W. (1992). Stable expression of functional human cytomegalovirus immediate-early proteins IE1 and IE2 in HeLa cells. Intervirology.

[92] Scheffner M., Werness B.A., Huibregtse J.M., Levine A.J., Howley P.M. (1990). The E6 oncoprotein encoded by human papillomavirus types 16 and 18 promotes the degradation of p53. Cell.

[93] Goldmacher V.S., Bartle L.M., Skaletskaya A., Dionne C.A., Kedersha N.L., Vater C.A., Han J.W., Lutz R.J., Watanabe S., Cahir McFarland E.D. (1999). A cytomegalovirus-encoded mitochondria-localized inhibitor of apoptosis structurally unrelated to Bcl-2. Proc. Natl. Acad. Sci. USA.

[94] Poncet D., Larochette N., Pauleau A.L., Boya P., Jalil A.A., Cartron P.F., Vallette F., Schnebelen C., Bartle L.M., Skaletskaya A. (2004). An anti-apoptotic viral protein that recruits Bax to mitochondria. J. Biol. Chem..

[95] Thomas M., Banks L. (1998). Inhibition of Bak-induced apoptosis by HPV-18 E6. Oncogene.

[96] Garnett T.O., Filippova M., Duerksen-Hughes P.J. (2006). Accelerated degradation of FADD and procaspase 8 in cells expressing human papilloma virus 16 E6 impairs TRAIL-mediated apoptosis. Cell Death Differ..

[97] Skaletskaya A., Bartle L.M., Chittenden T., McCormick A.L., Mocarski E.S., Goldmacher V.S. (2001). A cytomegalovirus-encoded inhibitor of apoptosis that suppresses caspase-8 activation. Proc. Natl. Acad. Sci. USA.

[98] Zhu H., Shen Y., Shenk T. (1995). Human cytomegalovirus IE1 and IE2 proteins block apoptosis. J. Virol..

[99] Bai Z., Li L., Wang B., Liu Z., Wang H., Yan Z., Qian D., Ding S., Song X. (2009). Effect of inducible expressed human cytomegalovirus immediate early 86 protein on cell apoptosis. Biosci. Biotechnol. Biochem..

[100] Gaiotti D., Chung J., Iglesias M., Nees M., Baker P.D., Evans C.H., Woodworth C.D. (2000). Tumor necrosis factor-alpha promotes human papillomavirus (HPV) E6/E7 RNA expression and cyclin-dependent kinase activity in HPV-immortalized keratinocytes by a ras-dependent pathway. Mol. Carcinog..

[101] Spangle J.M., Munger K. (2010). The human papillomavirus type 16 E6 oncoprotein activates mTORC1 signaling and increases protein synthesis. J. Virol..

[102] Spangle J.M., Munger K. (2013). The HPV16 E6 oncoprotein causes prolonged receptor protein tyrosine kinase signaling and enhances internalization of phosphorylated receptor species. PLoS Pathog..

[103] Hu G., Liu W., Hanania E.G., Fu S., Wang T., Deisseroth A.B. (1995). Suppression of tumorigenesis by transcription units expressing the antisense E6 and E7 messenger RNA (mRNA) for the transforming proteins of the human papilloma virus and the sense mRNA for the retinoblastoma gene in cervical carcinoma cells. Cancer Gene Ther..

[104] Boyer S.N., Wazer D.E., Band V. (1996). E7 protein of human papilloma virus-16 induces degradation of retinoblastoma protein through the ubiquitin-proteasome pathway. Cancer Res..

[105] McLaughlin-Drubin M.E., Park D., Munger K. (2013). Tumor suppressor p16INK4A is necessary for survival of cervical carcinoma cell lines. Proc. Natl. Acad. Sci. USA.

[106] Poma E.E., Kowalik T.F., Zhu L., Sinclair J.H., Huang E.S. (1996). The human cytomegalovirus IE1-72 protein interacts with the cellular p107 protein and relieves p107-mediated transcriptional repression of an E2F-responsive promoter. J. Virol..

[107] Zhang Z., Huong S.M., Wang X., Huang D.Y., Huang E.S. (2003). Interactions between human cytomegalovirus IE1-72 and cellular p107: Functional domains and mechanisms of up-regulation of cyclin E/cdk2 kinase activity. J. Virol..

[108] Valle Oseguera C.A., Spencer J.V. (2014). cmvIL-10 stimulates the invasive potential of MDA-MB-231 breast cancer cells. PLoS ONE.

[109] Oh S.T., Kyo S., Laimins L.A. (2001). Telomerase activation by human papillomavirus type 16 E6 protein: Induction of human telomerase reverse transcriptase expression through Myc and GC-rich Sp1 binding sites. J. Virol..

[110] Veldman T., Liu X., Yuan H., Schlegel R. (2003). Human papillomavirus E6 and Myc proteins associate in vivo and bind to and cooperatively activate the telomerase reverse transcriptase promoter. Proc. Natl. Acad. Sci. USA.

[111] Wang Y.W., Chang H.S., Lin C.H., Yu W.C. (2007). HPV-18 E7 conjugates to c-Myc and mediates its transcriptional activity. Int. J. Biochem. Cell Biol..

[112] Strååt K., Liu C., Rahbar A., Zhu Q., Liu L., Wolmer-Solberg N., Lou F., Liu Z., Shen J., Jia J. (2009). Activation of telomerase by human cytomegalovirus. J. Natl. Cancer Inst..

[113] Liu Y., Fan P., Yang Y., Xu C., Huang Y., Li D., Qing Q., Sun C., Zhou H. (2019). Human papillomavirus and human telomerase RNA component gene in cervical cancer progression. Sci. Rep..

[114] Liu H., Liu S., Wang H., Xie X., Chen X., Zhang X., Zhang Y. (2012). Genomic amplification of the human telomerase gene (hTERC) associated with human papillomavirus is related to the progression of uterine cervical dysplasia to invasive cancer. Diagn. Pathol..

[115] Toussaint-Smith E., Donner D.B., Roman A. (2004). Expression of human papillomavirus type 16 E6 and E7 oncoproteins in primary foreskin keratinocytes is sufficient to alter the expression of angiogenic factors. Oncogene.

[116] Wang N., Zhan T., Ke T., Huang X., Ke D., Wang Q., Li H. (2014). Increased expression of RRM2 by human papillomavirus E7 oncoprotein promotes angiogenesis in cervical cancer. Br. J. Cancer.

[117] Beisser P.S., Lavreysen H., Bruggeman C.A., Vink C. (2008). Chemokines and chemokine receptors encoded by cytomegaloviruses. Curr. Top. Microbiol. Immunol..

[118] Dumortier J., Streblow D.N., Moses A.V., Jacobs J.M., Kreklywich C.N., Camp D., Smith R.D., Orloff S.L., Nelson J.A. (2008). Human cytomegalovirus secretome contains factors that induce angiogenesis and wound healing. J. Virol..

[119] Maussang D., Langemeijer E., Fitzsimons C.P., Stigter-van Walsum M., Dijkman R., Borg M.K., Slinger E., Schreiber A., Michel D., Tensen C.P. (2009). The human cytomegalovirus-encoded chemokine receptor US28 promotes angiogenesis and tumor formation via cyclooxygenase-2. Cancer Res..

[120] Cheng Y.-M., Chou C.-Y., Hsu Y.-C., Chen M.-J., Wing L.-Y.C. (2012). The role of human papillomavirus type 16 E6/E7 oncoproteins in cervical epithelial-mesenchymal transition and carcinogenesis. Oncol. Lett..

[121] Yoshinouchi M., Yamada T., Kizaki M., Fen J., Koseki T., Ikeda Y., Nishihara T., Yamato K. (2003). In vitro and in vivo growth suppression of human papillomavirus 16-positive cervical cancer cells by E6 siRNA. Mol. Ther..

[122] Lea J.S., Sunaga N., Sato M., Kalahasti G., Miller D.S., Minna J.D., Muller C.Y. (2007). Silencing of HPV 18 oncoproteins With RNA interference causes growth inhibition of cervical cancer cells. Reprod. Sci..

[123] Vandermark E.R., Deluca K.A., Gardner C.R., Marker D.F., Schreiner C.N., Strickland D.A., Wilton K.M., Mondal S., Woodworth C.D. (2012). Human papillomavirus type 16 E6 and E 7 proteins alter NF-kB in cultured cervical epithelial cells and inhibition of NF-kB promotes cell growth and immortalization. Virology.

[124] Choudhury S., Woodworth C.D., Inamdar A., el-Beik T., DiPaolo J.A., Rosenthal L.J. (1992). Differences in retention and expression of transfected human cytomegalovirus Towne XbaI-E transforming fragment in human cervical and NIH 3T3 lines. Intervirology.

[125] Liu C., Han G., Li K., Si J., Liu S., Song G. (1999). Research on the oncogenesis of cervical epithelial cells co-induced by human papillomavirus and human cytomegalovirus. Zhonghua Bing Li Xue Za Zhi.

[126] Prieto-García E., Díaz-García C.V., García-Ruiz I., Agulló-Ortuño M.T. (2017). Epithelial-to-mesenchymal transition in tumor progression. Med. Oncol..

[127] Liu Y., Qian W., Zhang J., Dong Y., Shi C., Liu Z., Wu S. (2015). The indicative function of Twist2 and E-cadherin in HPV oncogene-induced epithelial-mesenchymal transition of cervical cancer cells. Oncol. Rep..

[128] Hellner K., Mar J., Fang F., Quackenbush J., Münger K. (2009). HPV16 E7 oncogene expression in normal human epithelial cells causes molecular changes indicative of an epithelial to mesenchymal transition. Virology.

[129] Nehme Z., Pasquereau S., Haidar Ahmad S., El Baba R., Herbein G. (2022). Polyploid giant cancer cells, EZH2 and Myc upregulation in mammary epithelial cells infected with high-risk human cytomegalovirus. EBioMedicine.

[130] Steinbach A., Riemer A.B. (2018). Immune evasion mechanisms of human papillomavirus: An update. Int. J. Cancer.

[131] Noriega V., Redmann V., Gardner T., Tortorella D. (2012). Diverse immune evasion strategies by human cytomegalovirus. Immunol. Res..

[132] Takeuchi O., Akira S. (2009). Innate immunity to virus infection. Immunol. Rev..

[133] De Nardo D. (2015). Toll-like receptors: Activation, signalling and transcriptional modulation. Cytokine.

[134] Westrich J.A., Warren C.J., Pyeon D. (2017). Evasion of host immune defenses by human papillomavirus. Virus Res..

[135] Li S., Xie Y., Yu C., Zheng C., Xu Z. (2024). The battle between host antiviral innate immunity and immune evasion by cytomegalovirus. Cell. Mol. Life Sci..

[136] Hasan U.A., Bates E., Takeshita F., Biliato A., Accardi R., Bouvard V., Mansour M., Vincent I., Gissmann L., Iftner T. (2007). TLR9 expression and function is abolished by the cervical cancer-associated human papillomavirus type 16. J. Immunol..

[137] Pacini L., Savini C., Ghittoni R., Saidj D., Lamartine J., Hasan U.A., Accardi R., Tommasino M. (2015). Downregulation of Toll-Like Receptor 9 Expression by Beta Human Papillomavirus 38 and Implications for Cell Cycle Control. J. Virol..

[138] Daud I.I., Scott M.E., Ma Y., Shiboski S., Farhat S., Moscicki A.B. (2011). Association between toll-like receptor expression and human papillomavirus type 16 persistence. Int. J. Cancer.

[139] Park A., Ra E.A., Lee T.A., Choi H.J., Lee E., Kang S., Seo J.Y., Lee S., Park B. (2019). HCMV-encoded US7 and US8 act as antagonists of innate immunity by distinctively targeting TLR-signaling pathways. Nat. Commun..

[140] Landais I., Pelton C., Streblow D., DeFilippis V., McWeeney S., Nelson J.A. (2015). Human Cytomegalovirus miR-UL112-3p Targets TLR2 and Modulates the TLR2/IRAK1/NFkappaB Signaling Pathway. PLoS Pathog..

[141] Harwani S.C., Lurain N.S., Zariffard M.R., Spear G.T. (2007). Differential inhibition of human cytomegalovirus (HCMV) by toll-like receptor ligands mediated by interferon-beta in human foreskin fibroblasts and cervical tissue. Virol. J..

[142] Ronco L.V., Karpova A.Y., Vidal M., Howley P.M. (1998). Human papillomavirus 16 E6 oncoprotein binds to interferon regulatory factor-3 and inhibits its transcriptional activity. Genes Dev..

[143] Liu T., Zhang L., Joo D., Sun S.-C. (2017). NF-κB signaling in inflammation. Signal Transduct. Target. Ther..

[144] Pfeffer L.M. (2011). The role of nuclear factor kappaB in the interferon response. J. Interferon Cytokine Res..

[145] Spitkovsky D., Hehner S.P., Hofmann T.G., Moller A., Schmitz M.L. (2002). The human papillomavirus oncoprotein E7 attenuates NF-kappa B activation by targeting the Ikappa B kinase complex. J. Biol. Chem..

[146] Hancock M.H., Hook L.M., Mitchell J., Nelson J.A. (2017). Human Cytomegalovirus MicroRNAs miR-US5-1 and miR-UL112-3p Block Proinflammatory Cytokine Production in Response to NF-kappaB-Activating Factors through Direct Downregulation of IKKalpha and IKKbeta. mBio.

[147] Mathers C., Schafer X., Martinez-Sobrido L., Munger J. (2014). The human cytomegalovirus UL26 protein antagonizes NF-kappaB activation. J. Virol..

[148] Havard L., Rahmouni S., Boniver J., Delvenne P. (2005). High levels of p105 (NFKB1) and p100 (NFKB2) proteins in HPV16-transformed keratinocytes: Role of E6 and E7 oncoproteins. Virology.

[149] Patel D., Huang S.M., Baglia L.A., McCance D.J. (1999). The E6 protein of human papillomavirus type 16 binds to and inhibits co-activation by CBP and p300. EMBO J..

[150] Huang S.M., McCance D.J. (2002). Down regulation of the interleukin-8 promoter by human papillomavirus type 16 E6 and E7 through effects on CREB binding protein/p300 and P/CAF. J. Virol..

[151] Bonjardim C.A., Ferreira P.C.P., Kroon E.G. (2009). Interferons: Signaling, antiviral and viral evasion. Immunol. Lett..

[152] Raikhy G., Woodby B.L., Scott M.L., Shin G., Myers J.E., Scott R.S., Bodily J.M. (2019). Suppression of Stromal Interferon Signaling by Human Papillomavirus 16. J. Virol..

[153] Reiser J., Hurst J., Voges M., Krauss P., Munch P., Iftner T., Stubenrauch F. (2011). High-risk human papillomaviruses repress constitutive kappa interferon transcription via E6 to prevent pathogen recognition receptor and antiviral-gene expression. J. Virol..

[154] Wang H., Peng W., Wang J., Zhang C., Zhao W., Ran Y., Yang X., Chen J., Li H. (2023). Human Cytomegalovirus UL23 Antagonizes the Antiviral Effect of Interferon-gamma by Restraining the Expression of Specific IFN-Stimulated Genes. Viruses.

[155] Bermudez-Morales V.H., Peralta-Zaragoza O., Alcocer-Gonzalez J.M., Moreno J., Madrid-Marina V. (2011). IL-10 expression is regulated by HPV E2 protein in cervical cancer cells. Mol. Med. Rep..

[156] Bermudez-Morales V.H., Gutierrez L.X., Alcocer-Gonzalez J.M., Burguete A., Madrid-Marina V. (2008). Correlation between IL-10 gene expression and HPV infection in cervical cancer: A mechanism for immune response escape. Cancer Investig..

[157] Bhairavabhotla R.K., Verm V., Tongaonkar H., Shastri S., Dinshaw K., Chiplunkar S. (2007). Role of IL-10 in immune suppression in cervical cancer. Indian J. Biochem. Biophys..

[158] Dziurzynski K., Wei J., Qiao W., Hatiboglu M.A., Kong L.Y., Wu A., Wang Y., Cahill D., Levine N., Prabhu S. (2011). Glioma-associated cytomegalovirus mediates subversion of the monocyte lineage to a tumor propagating phenotype. Clin. Cancer Res..

[159] Gruber S.G., Gloria Luciani M., Grundtner P., Zdanov A., Gasche C. (2008). Differential signaling of cmvIL-10 through common variants of the IL-10 receptor 1. Eur. J. Immunol..

[160] Stropes M.P., Miller W.E. (2004). Signaling and regulation of G-protein coupled receptors encoded by cytomegaloviruses. Biochem. Cell Biol..

[161] Bodaghi B., Jones T.R., Zipeto D., Vita C., Sun L., Laurent L., Arenzana-Seisdedos F., Virelizier J.L., Michelson S. (1998). Chemokine sequestration by viral chemoreceptors as a novel viral escape strategy: Withdrawal of chemokines from the environment of cytomegalovirus-infected cells. J. Exp. Med..

[162] Vieira J., Schall T.J., Corey L., Geballe A.P. (1998). Functional analysis of the human cytomegalovirus US28 gene by insertion mutagenesis with the green fluorescent protein gene. J. Virol..

[163] Torres-Poveda K., Bahena-Roman M., Madrid-Gonzalez C., Burguete-Garcia A.I., Bermudez-Morales V.H., Peralta-Zaragoza O., Madrid-Marina V. (2014). Role of IL-10 and TGF-beta1 in local immunosuppression in HPV-associated cervical neoplasia. World J. Clin. Oncol..

[164] Xu Q., Wang S., Xi L., Wu S., Chen G., Zhao Y., Wu Y., Ma D. (2006). Effects of human papillomavirus type 16 E7 protein on the growth of cervical carcinoma cells and immuno-escape through the TGF-beta1 signaling pathway. Gynecol. Oncol..

[165] Baritaki S., Sifakis S., Huerta-Yepez S., Neonakis I.K., Soufla G., Bonavida B., Spandidos D.A. (2007). Overexpression of VEGF and TGF-beta1 mRNA in Pap smears correlates with progression of cervical intraepithelial neoplasia to cancer: Implication of YY1 in cervical tumorigenesis and HPV infection. Int. J. Oncol..

[166] Fan D.M., Tian X.Y., Wang R.F., Yu J.J. (2014). The prognosis significance of TGF-beta1 and ER protein in cervical adenocarcinoma patients with stage Ib~IIa. Tumour Biol..

[167] Peralta-Zaragoza O., Bermudez-Morales V., Gutierrez-Xicotencatl L., Alcocer-Gonzalez J., Recillas-Targa F., Madrid-Marina V. (2006). E6 and E7 oncoproteins from human papillomavirus type 16 induce activation of human transforming growth factor beta1 promoter throughout Sp1 recognition sequence. Viral Immunol..

[168] Michelson S., Alcami J., Kim S.J., Danielpour D., Bachelerie F., Picard L., Bessia C., Paya C., Virelizier J.L. (1994). Human cytomegalovirus infection induces transcription and secretion of transforming growth factor beta 1. J. Virol..

[169] Kwon Y.J., Kim D.J., Kim J.H., Park C.G., Cha C.Y., Hwang E.S. (2004). Human cytomegalovirus (HCMV) infection in osteosarcoma cell line suppresses GM-CSF production by induction of TGF-beta. Microbiol. Immunol..

[170] Yoo Y.D., Chiou C.J., Choi K.S., Yi Y., Michelson S., Kim S., Hayward G.S., Kim S.J. (1996). The IE2 regulatory protein of human cytomegalovirus induces expression of the human transforming growth factor beta1 gene through an Egr-1 binding site. J. Virol..

[171] Campo M.S., Graham S.V., Cortese M.S., Ashrafi G.H., Araibi E.H., Dornan E.S., Miners K., Nunes C., Man S. (2010). HPV-16 E5 down-regulates expression of surface HLA class I and reduces recognition by CD8 T cells. Virology.

[172] Kim D.H., Kim E.M., Lee E.H., Ji K.Y., Yi J., Park M., Kim K.D., Cho Y.Y., Kang H.S. (2011). Human papillomavirus 16E6 suppresses major histocompatibility complex class I by upregulating lymphotoxin expression in human cervical cancer cells. Biochem. Biophys. Res. Commun..

[173] Zimmermann C., Kowalewski D., Bauersfeld L., Hildenbrand A., Gerke C., Schwarzmuller M., Le-Trilling V.T.K., Stevanovic S., Hengel H., Momburg F. (2019). HLA-B locus products resist degradation by the human cytomegalovirus immunoevasin US11. PLoS Pathog..

[174] Maaßen F., Le-Trilling V.T.K., Betke L., Bracht T., Schuler C., Bayer M., Belter A., Becker T., Katschinski B., Frappier L. (2023). The human cytomegalovirus-encoded pUS28 antagonizes CD4+ T-cell recognition by targeting CIITA. bioRxiv.

